# Short- and Medium-Chain Chlorinated Paraffins in the Sediment of the East China Sea and Yellow Sea: Distribution, Composition, and Ecological Risks

**DOI:** 10.3390/toxics11070558

**Published:** 2023-06-26

**Authors:** Xiaoying Li, Haiqiang Guo, Jianyao Hong, Yuan Gao, Xindong Ma, Jiping Chen

**Affiliations:** 1 College of Environmental Science and Engineering, Dalian Maritime University, Dalian 116026, China; 2 CAS Key Laboratory of Separation Sciences for Analytical Chemistry, Dalian Institute of Chemical Physics, Chinese Academy of Sciences, 457 Zhongshan Road, Shahekou District, Dalian 116023, China; 3 State Environmental Protection Key Laboratory of Coastal Ecosystem, National Marine Environmental Monitoring Center, Dalian 116023, China

**Keywords:** chlorinated paraffin, marine sediments, spatial variations, ecological risk

## Abstract

Chlorinated paraffins (CPs), a class of complex mixtures synthesized from polychlorinated n-alkanes, are widely used as flame retardants, plasticizers, lubricant additives, coolants, metalworking cutting fluids, and sealants. This study investigated the spatial distribution, the potential pollution sources, and ecological risk of 24 short-chain CPs (SCCPs) and 24 medium-chain CPs (MCCPs) from 29 surface marine sediment samples from the East China Sea and Yellow Sea in September 2019. All of the 48 CPs were detected. The concentrations of SCCPs and MCCPs ranged from 0.703 to 13.4 ng/g dw and 0.0936 to 4.19 ng/g dw, respectively. C_10_ congeners showed the highest abundancy. The median concentrations of the SCCPs and MCCPs declined gradually with carbon atoms and chlorine atoms, except for Cl_5_ congeners. Spatial variations showed that all CP congeners in the East China Sea were larger than in the Yellow Sea and displayed a point-source-type distribution, which is consistent with the industrial park distribution. Although the potential ecological risk was at a relatively low level, bioaccumulation and trophic magnification could amplify the risk to marine organisms. Our results provide data support and theoretical assistance for SCCP and MCCP pollution control and sewage outlets in the East China Sea and Yellow Sea.

## 1. Introduction

Chlorinated paraffins (CPs) are a class of complex mixtures synthesized from polychlorinated n-alkanes, usually containing 10–30 carbon atoms [1]. Because of their good thermal and chemical stability, CPs are widely used as flame retardants, plasticizers, lubricant additives, coolants, metalworking cutting fluids, and sealants [1,2,3]. Although China is the largest producer and consumer of CPs in the world, with an annual production of 1.12 million tons in 2013 [4], information related to their environmental occurrence and distribution remains limited. As a result of long-term excess industrial application, a large quantity of CPs has been released into the environment and, thus, widely detected in water, atmosphere, soil, sediment, plants, animals, and even humans [1,4,5,6]. CPs, especially short-chain CPs (SCCPs, C_10–13_), have attracted attention due to their sustained release, persistence, long-range transport, bioaccumulation, and toxic characteristics; therefore, they have been listed as substances of very high concern by the Stockholm Convention and controlled globally since 2017. Medium-chain CPs (MCCPs, C_14–17_), although not categorized as substances of very high concern, are listed on the “Community Rolling Action Plan” as a Risk Management Option. Analysis methods must be developed to determine the potential bioaccumulative and reprotoxic properties of MCCPs. Consequently, the sources, migration path, anthropogenic contamination, and fate in the marine environment of SCCPs and MCCPs need to be explored to reduce their potential environmental and health risks.

Although CPs have been transported worldwide, marine sediment is an important pool of them, second only to industrial sediments in terms of concentration, which is supplied by the atmosphere, wet or dry precipitation, and river sewage outlets. After CPs are introduced into the aquatic environment from estuaries and coasts, they tend to be adsorbed by organic and inorganic particles and into sediments, thereby settling to the bottom. The concentration of CPs in marine sediments usually increases with time, but it decreases with distance as they move further away from the coast of the East China Sea, Bohai Sea, and Yellow Sea [7,8]. Therefore, coastal sediment is the main reservoir of anthropogenic CPs and a reliable record of past CP pollution. Moreover, CPs in estuarine and coastal environments also affect a large number of marine organisms, not only rendering them an ecological risk, but also a risk to the health of coastal residents.

It has been reported that due to continuous input from surrounding sewage treatment plants, CP pollution is serious in the East China Sea, which has the largest fishing area in China [9,10]. For this reason, previous studies have detected CPs in various media, such as water, sediment, and marine organisms [6,8]. Regional and economic differences are critical factors that determine CP exposure. Moreover, differences in CPs have also been attributed to land-based atmospheric sedimentation, emissions, major surface currents, and mud areas. Thus, exploring the origin and potential influencing factors plays a crucial role in the environmental fate, pollution control, and health of residents.

This study analyzed SCCPs and MCCPs in sediment collected from the Yangtze River Estuary in 2019 to reveal the distribution and homologue group abundance patterns. The specific objectives of this study were (1) to gain an overview of the concentration levels and congener profiles in sediments of this area; (2) to explore the spatial distribution and the potential pollution sources of SCCPs and MCCPs in sediments; and (3) to evaluate the ecological risk to sediments. This investigation could improve the fundamental understanding of the environmental occurrence and fate of CPs and provide a scientific basis for the marine geochemical study of SCCPs and MCCPs in the Yangtze River estuary, which may be beneficial when assessing the risk posed by CPs to both the local ecology system and people living in this coastal region.

## 2. Materials and Methods

### 2.1. Solvents and Chemical Standards

Three commercial standards for SCCPs (with chlorine contents of 51.5%, 55.5%, and 63.0%, 100 µg/mL) and MCCPs (with chlorine contents of 42%, 52%, and 57%, 100 µg/mL) were purchased from Dr. Ehrenstorfer GmbH (Augsburg, Germany). The stable isotope-labeled internal *trans*-chlordan (^13^C_10_ *trans*-chlordan, 100 µg/L in nonane) and hexachlorobenzene (100 µg/L) were purchased from Cambridge Isotope Laboratories (Andover, MA, USA). Dichloromethane, n-hexane, and n-nonane were purchased from J.T. Baker (Radnor, PA, USA).

### 2.2. Studied Area and Sample Collection

**Figure 1:**
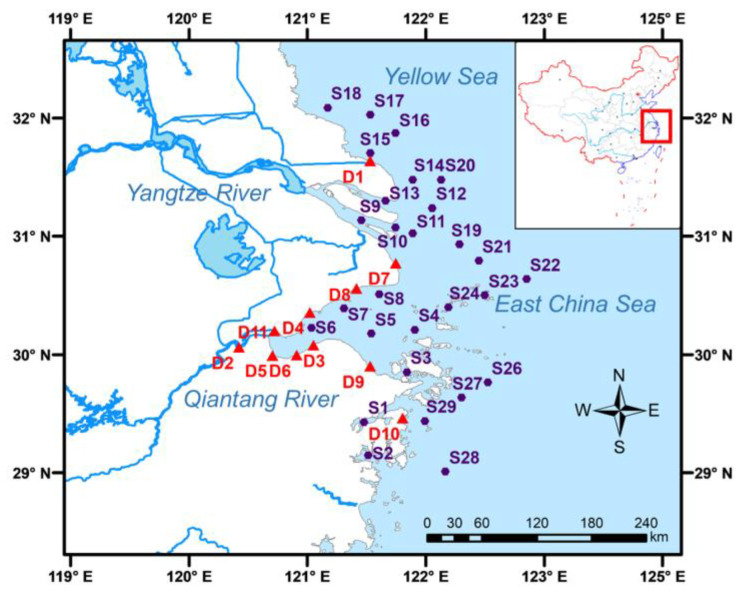
The sampling sites and sewage outlets in the East China Sea and Yellow Sea, China.

In order to comprehensively understand the contamination status of CPs in the East China Sea and the Yellow Sea, surface marine sediment samples (0–5 cm) were collected in September 2019. A total of 29 sampling locations were selected as shown in Figure 1, including 5 in the Yellow Sea (S14, S15, S16, S17, and S18) and 24 in the East China Sea (S1, S2, S3, S4, S5, S6, S7, S8, S9, S10, S11, S12, S13, S19, S20, S21, S22, S23, S24, S25, S26, S27, S28, and S29). Moreover, 10 (D2, D3, D4, D5, D6, D7, D8, D9, D10, and D11) and 1 (D1) sewage outlets were found in the East China Sea and the Yellow Sea, respectively, and are shown in Figure 1. The sediment samples were freeze-dried with a vacuum freeze dryer (Labconco Corporation, Kansas City, MO, USA) and then homogenized and stored at −20 °C until extraction.

### 2.3. Sample Extraction and Cleanup

In this study, the extraction and clean-up procedures were modified based on previous studies [11]. Briefly, 10.0 g of the sediment spiked with 10 µL of 1 × 10^4^ ng/mL ^13^C_10_-*trans*-chlordane was extracted using 40.0 mL of n-hexane/dichloromethane (1/1, *v*/*v*) under ultrasound (40 kHz) for 15 min and centrifugated (2000 rpm/min, 5 min) to obtain the supernatant. This process was repeated thrice to ensure sufficient extraction. Next, the extract was concentrated to approximately 1.5 mL and then passed through an acidified silica–florisil composite column (inside diameter: 26 mm) containing, from bottom to top, 5.0 g of anhydrous sodium sulfate, 2.0 g of activated neutral silica gel, 4.5 g of 44.0% acid silica gel, and 6.0 g of anhydrous sodium sulfate. The column was preconditioned with 30 mL of dichloromethane and 50 mL of n-hexane for removing impurities. After the loading of the entire sample mixture (approximately 1.5 mL), the column was washed with 30 mL of n-hexane. Finally, the cartridges were eluted with 50 mL of n-hexane/dichloromethane (1/1, *v*/*v*) and 50 mL of n-hexane/dichloromethane (1/2, *v*/*v*). The collected eluent was evaporated to nearly 1.5 mL with a rotary evaporator (R–205 Type, BUCHI Labortechnik AG, Flawil, Switzerland). Then, the 1.5 mL solution was evaporated nearly to dryness in autosampler vials with a gentle stream of pure nitrogen (99.9%). In total, 10 µL of 1 ng/mL hexachlorobenzene was added as an injection internal standard and vortexed for further analysis.

### 2.4. Instrumental Analysis

Instrumental analysis was performed using gas chromatography–mass spectrometry (GC-MS) and electron capture negative ionization (ECNI) (QP2010, Shimadzu, Nakagyo-ku, Kyoto, Japan) coupled with a capillary DB-5 column (15 m × 0.25 mm i.d. × 0.25 μm, CNW ANPEL, Shanghai, China). The injection volume was 1 μL for SCCPs and MCCPs, respectively. Each 1 μL sample was injected in the splitless mode, and the injector temperature was 280 °C. A constant flow (1 mL/min) of high-purity helium (99.9%) was used as a carrier gas. The oven temperature started at 100 °C (held for 2 min), followed by heating to 160 °C at 20 °C/min (held for 2 min), then was increased to 310 °C at 30 °C/min (held for 15 min). Detailed information, including the percentage of Cl in the molecular formula, quantitative ion, and confirmation ion, and the relative abundance used to determine SCCPs, MCCPs, ^13^C_10_ *trans*-chlordan, and hexachlorobenzene, is listed in Table S1. The temperature of the ion source and the transfer line were kept at 200 °C and 280 °C, respectively. The reaction gas was methane gas with a flow velocity of 2 mL/min along with a 5 min solvent delay. The two most abundant isotopes of the [M − HCl]^−^ cluster were selected for quantitative and qualitative ions. The [M − Cl]^−^ and [M]^−^ ions were selected as the monitoring ions.

### 2.5. Quality Assurance and Quality Control

To eliminate background interference as much as possible, all glass containers were washed 3 times with high-purity dichloromethane and 3 times with high-purity hexane before use. To improve the instrument sensitivity and minimize the interference of MCCP congeners, each sample was injected four times in the selected ion monitoring (SIM) mode to monitor the SCCP ions of C_10_, C_11_, C_12_, and C_13_, respectively. One blank sample was added for every 10 samples to detect the background contamination. The extraction, cleanup, and instrumental analysis of the blanks were performed separately according to the above treatment. The standard deviation was less than 15.0%, and the method detection limit (MDL) was the mean value of the blank plus 3 times the standard deviation after 6 sample replicates. The recoveries were the ratios of ^13^C_10_ *trans*-chlordan and hexachlorobenzene in the sediment samples to the ratios of ^13^C_10_ *trans*-chlordan in the standard solutions. The recoveries for SCCPs and MCCPs were 69.9–120% and 61.4–102%, respectively; the MDL for SCCPs and MCCPs was 0.460 and 0.00800 ng/g dw, respectively.

### 2.6. Data Analysis

Data, statistical analyses, and figure drawing were performed using SPSS (version 22.0; Chicago, IL, USA) and OriginLab OriginPro 8.5 (Northampton, MA, USA). The significance of the differences between data was tested using one-way ANOVA (normal distribution) or the Mann–Whitney U test (non-normal distribution). Linear dependence was determined using Spearman’s rank correlation analysis. The statistical value of *p* < 0.05 was considered significant for the above statistical analysis method.

## 3. Results and Discussion

### 3.1. Concentrations and Profiles of SCCPs and MCCPs in Sediments

We identified 24 SCCP and 24 MCCP congeners in the tested sediments. These CPs were all detected in 29 sediments from the East China Sea and Yellow Sea. Their total concentrations ranged from 0.796 to 17.6 ng/g dw, with a median value of 1.94 ng/g dw. The SCCPs ranged from 0.703 to 13.4 ng/g dw, with the median value of 1.70 ng/g dw (Table 1). The MCCPs ranged from 0.0936 to 4.19 ng/g dw, with a median value of 0.212 ng/g dw. The median concentration of SCCPs was eight times that of MCCPs. The highest concentrations all occurred in Ninghai County, Ningbo, Zhejiang Province (S1), located in narrow and semi-enclosed bays of Xiangshan Harbor. To the best of our knowledge, this area is adjacent to various industrial parks of Fenghua District and Xiangshan County (Ningbo, Zhejiang Province). Moreover, a significant positive correlation (*p* < 0.001) was observed among SCCP and MCCP congeners (Figure S1), which was ascribed to them having the same source and similar transformation pathways.

The SCCPs and MCCPs in marine sediments in this study were all at a lower level compared to those in previous reports (Table S2). For the most commonly detected SCCPs, the concentration (range: 0.703–13.4 ng/g dw) in this study was one or two orders of magnitude lower than that in marine sediments from Laizhou Bay (5.10–22.0 ng/g dw) [12], the Yangtze River Estuary and the adjacent East China Sea (2.85–94.7 ng/g dw) [13], as well as the Firth of Clyde in Scotland (0.400–69.0 ng/g dw) [14], the South China Coast (133–716 ng/g dw) [15], Shandong Peninsula (<MDL–453 ng/g dw) [5], the East China Sea in 2010–2011 (9.00–37.2 ng/g dw) [16], and East China Sea in 2019 (89.6–351 ng/g dw) [17]. In a study of Shandong Peninsula, SCCPs were undetected in partial sediment samples [5]. For MCCPs, the concentration (range: 0.0936–4.19 ng/g dw) in this study was lower than the middle and lower reaches of Laizhou Bay (2.20–63.0 ng/g dw) [12], the Yangtze River Estuary and adjacent East China Sea (3.33–77.8 ng/g dw) [13], and the South China Coast (Guangdong, Fujian, Guangxi, and Hainan) (103–4160 ng/g dw) [15]. There was a relatively low CP concentration in this study, especially for SCCPs. With a significant exponential trend in production of CPs in China since 2003, the SCCP concentrations showed a gradually increasing trend from the deeper segments (36–68 cm) to the upper segments (0–32 cm), but no obvious trend was observed for the MCCPs in the Pearl River Delta [18]. Sediments in different regions showed various CP levels, which were attributed to industrial pollution sources, the sampling depth, sedimentation rate, the texture of the sediment, ocean currents, physicochemical properties, and policies.

In order to evaluate the ecological risk of SCCPs in sediments, the Federal Environmental Quality Guidelines for chlorinated alkanes developed by Environment Canada were used [19]. Based on the Federal Sediment Quality Guidelines, the risks that SCCPs and MCCPs pose to sediment-dwelling and pelagic animals were also classified into three risk levels, respectively. For SCCPs, these three risk levels are: ≤1800 ng/g dw, low risk; >1800 and ≤18,000 ng/g dw, medium risk; and >18,000 ng/g dw, high risk. For MCCPs, these levels are: ≤5400 ng/g dw, low risk; >5400 and ≤54,000 ng/g dw, medium risk; >54,000 ng/g dw, high risk.

**Figure 2:**
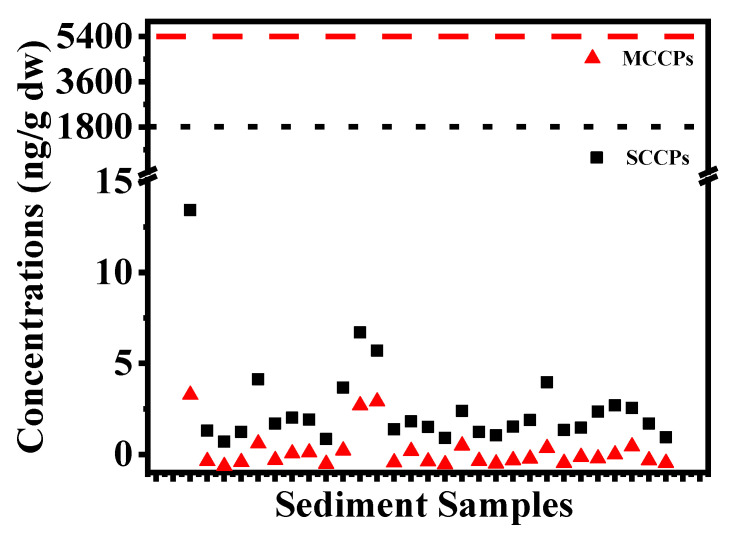
The concentrations of SCCPs and MCCPs in sediments compared with chlorinated alkanes in the Federal Environmental Quality Guidelines.

In the East China Sea and Yellow Sea, the SCCP and MCCP levels in all sediments were far less than 1800 and 5400 ng/g dw (Figure 2), indicating that they posed a low ecological risk in sediments from all sampling sites. The low CP level results were similar to the previous research on CPs in sediments of Yangtze River Estuary and adjacent East China Sea [13]. However, trophic magnification of SCCPs was observed in the marine food web. In our previous study, bioaccumulation and trophic magnification of SCCPs were found with 65.0–541 ng/g dw in sediment but with 86.0–4400 ng/g ww in organisms [20]. Moreover, trophic magnification of SCCPs and concentrations which surpassed the Federal Fish Tissue Guidelines were observed in the marine food web from the East China Sea [6]. These studies all illustrated the potential adverse effects on marine organisms with a lower environmental concentration. A long-term investigation of CPs in sediments is recommended to reduce accumulation and uncertainty in regions and achieve seafood safety and biotransformation.

### 3.2. Congener Compositional Profiles, Sources, and Environmental Behaviors of SCCPs and MCCPs in Sediments

The concentrations of SCCPs in the sediments were far higher than those of MCCPs. C_10_ and C_11_ congeners were the main SCCPs, with a median relative abundance of 43.5% and 24.2% (Figure S2), respectively. Similar to multiple previous studies [12,13], C_14_ congeners and C_15_ congeners were the main MCCPs, with a median relative abundance of 48.8% and 25.0%, respectively. Similar to carbon atoms, the range of median relative abundance for chlorine concentrations was from 4.79% (Cl_10_ congeners) to 34.7% (Cl_6_ congeners) for SCCPs and from 1.64% (Cl_10_ congeners) to 23.2% for MCCPs (Cl_5_ congeners) (Table S3 and Figure S3). Similar to previous studies [17], the dominant chlorine congener groups were Cl_5_, Cl_6_- and Cl_7_ congeners.

The ratio of MCCPs to SCCPs (MCCPs:SCCPs) is an important index for discussing direct emission sources [12,18]. The median MCCPs:SCCPs ratio was calculated to be 12.6% in the range of 1.93–42.4%, which indicated that there was no direct source of CPs in this area. There was a similar phenomenon in previous studies; long-range transport was thought to be the major pathway for CPs into the sediments [21]. In contrast, the concentration of MCCPs was found to be higher than that of SCCPs in some CP manufacturing zones, such as Laizhou Bay [12]. CP factories may be located far away from the study area. The distribution of CPs is influenced by the geographical location, river conditions, the sea area, and human activities in the surrounding area, as well as the different physicochemical properties of SCCPs and MCCPs, migration velocity, transformation, and accumulation in environmental samples.

### 3.3. Spatial Distribution of SCCPs and MCCPs in the Sediments

**Figure 3:**
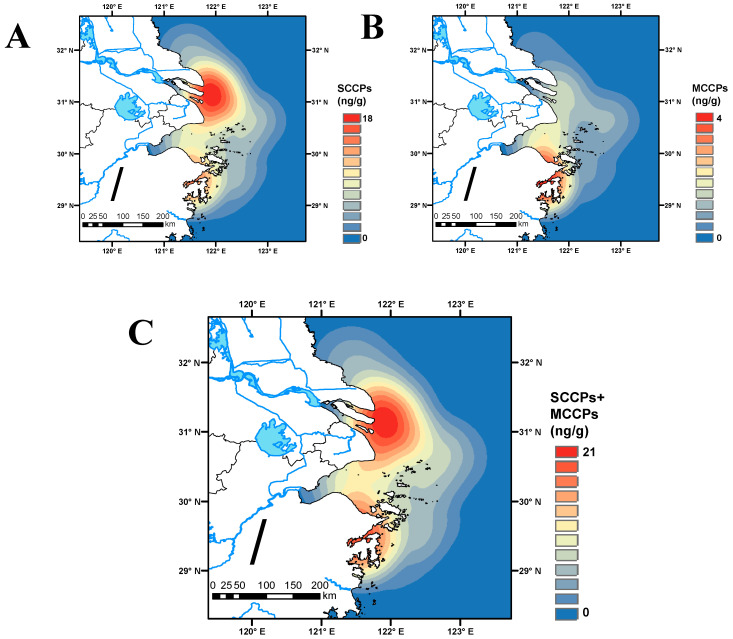
Spatial distributions of SCCPs (**A**) and MCCPs (**B**), and the total SCCP and MCCP (**C**) concentrations in marine sediments of the study area in the East China Sea and Yellow Sea.

The concentrations of SCCPs and MCCPs (median: 1.79 and 0.229 ng/g dw, respectively) in the East China Sea were far higher than those in the Yellow Sea (1.52 and 0.121 ng/g dw, respectively) (Figure S4). The actual sample size and sampling time limited the comparison between sea areas. The spatial distribution of the total concentration of SCCPs and MCCPs in the East China Sea and Yellow Sea displayed a point-source-type distribution from coastal land to the ocean, which decreased vastly from Ninghai County, Ningbo, Zhejiang Province (S1) to Hengshan District, Chongming District, Shanghai (S11 and S12) (Figure 3). For S1, multiple industrial parks are spread all over the counties and districts of Ningbo, Zhejiang Province. As a typical semi-enclosed bay, industrial emissions accelerated the speed and degree of pollution. For S11 and S12, the sampling sites are close to the mud areas reported in the Yangtze Estuary of a previous study [8]. Salinity gradient energy promoted the adsorption of organic pollutants in fine suspended particles at the river–sea junction.

The spatial distribution of SCCPs was similar to the total concentration of SCCPs and MCCPs (Figure 3). For MCCPs, the sampling site of S1 and S5 was higher than other sampling points. Two sewage outlets (D3 and D9) were found near S5. An oil refinery plant is located in S5 [22]. Moreover, Xiangshan Harbor (Ningbo, Zhejiang Province) has been reported as the most significant location, containing paper mills, area chemical companies, and a coal-powered plant [22,23]. Overall, the intensity of human activity near important coastal bays directly increases CP levels, and closed areas are worth paying attention to.

## 4. Conclusions

In summary, this study reported SCCPs and MCCPs based on 29 marine sediment samples from the East China Sea and Yellow Sea, investigating their spatial distribution, potential pollution sources, and ecological risks. The concentrations of SCCPs and MCCPs ranged from 0.703 to 13.4 ng/g dw and 0.0936 to 4.19 ng/g dw, respectively. Among 48 CP congeners, C_10_ congeners were the most abundant, and the median concentrations of SCCPs and MCCPs declined gradually with carbon atoms and chlorine atoms, except for Cl_5_ congeners. Spatial variations showed that all CP congeners in the East China Sea were larger than in the Yellow Sea and displayed point-source-type distributions, which is consistent with the industrial park distribution. Although the potential ecological risk is at a low level, bioaccumulation and trophic magnification can amplify the risk to marine organisms. Therefore, focusing on regional CP pollution is essential, especially in typical semi-enclosed bays with industrial parks. Although this study provided data support and theoretical assistance for pollution and discharge control, the small actual sample size limited comparison between sea areas and studying the environmental fate, resulting in uncertainty in the results. This study provides basic data for understanding the environmental behavior and fate of SCCPs and MCCPs.

## Figures and Tables

**Table 1:** Concentration of SCCPs and MCCPs (ng/g dw) and relative abundance of individual homolog (%) in marine sediment samples in the East China Sea and Yellow Sea.

Samples	SCCPs (ng/g dw)	C_10_ (%)	C_11_ (%)	C_12_ (%)	C_13_ (%)	MCCPs (ng/g dw)	C_14_ (%)	C_15_ (%)	C_16_ (%)	C_17_ (%)
S1	13.4	29.2	22.4	23.5	24.9	4.19	42.8	27.5	15.1	14.5
S2	1.30	44.3	19.0	17.6	19.1	0.297	45.0	29.7	13.0	12.3
S3	0.703	45.6	19.3	16.6	18.4	0.0936	43.4	31.9	12.9	11.7
S4	1.24	42.0	19.2	18.5	20.3	0.152	56.7	24.4	10.3	8.62
S5	4.10	35.4	14.0	17.5	33.1	1.75	69.1	20.7	4.64	5.51
S6	1.70	36.9	28.4	18.0	16.6	0.333	43.9	24.3	16.6	15.3
S7	2.01	47.8	21.2	15.3	15.6	0.253	52.4	24.9	11.3	11.4
S8	1.92	52.9	19.4	12.7	15.0	0.232	58.7	24.8	8.18	8.32
S9	0.838	51.5	23.0	13.4	12.1	0.971	44.0	31.6	14.1	10.3
S10	3.68	30.2	20.9	31.7	17.3	0.675	47.7	18.2	8.72	25.4
S11	6.70	50.7	20.6	14.0	14.7	0.611	55.8	25.0	8.51	10.7
S12	5.71	62.9	24.5	8.26	4.25	0.110	38.3	40.0	14.0	7.68
S13	1.38	37.0	31.6	12.2	19.3	0.164	42.1	28.5	18.0	11.4
S14	1.82	59.8	24.5	6.95	8.81	0.121	38.4	29.6	12.0	20.0
S15	1.52	36.7	27.5	18.9	16.9	0.212	48.7	23.4	15.2	12.7
S16	0.903	45.7	26.1	14.0	14.2	0.108	49.4	25.7	15.0	9.85
S17	2.39	57.2	27.3	8.11	7.38	0.109	42.4	35.1	15.2	7.32
S18	1.24	47.0	19.7	12.8	20.5	0.357	62.9	24.0	6.97	6.08
S19	1.04	43.5	29.0	14.4	13.1	0.115	37.1	33.8	17.6	11.5
S20	1.52	40.8	29.7	15.3	14.2	0.133	38.1	29.2	20.4	12.3
S21	1.89	37.1	28.2	18.0	16.7	0.190	54.3	23.0	12.9	9.84
S22	3.97	31.0	19.3	15.6	34.1	0.941	69.0	20.1	5.97	4.94
S23	1.34	36.1	26.3	19.4	18.1	0.208	51.8	24.6	13.1	10.6
S24	1.47	53.7	24.2	11.0	11.2	0.0960	48.8	29.2	11.7	10.3
S25	2.35	30.1	13.0	14.3	42.7	0.873	73.4	18.2	4.07	4.35
S26	2.69	33.7	25.5	19.0	21.8	0.536	63.3	19.6	9.45	7.60
S27	2.56	51.6	25.4	10.9	12.0	0.227	58.7	23.1	10.5	7.66
S28	1.69	36.1	30.1	16.4	17.5	0.240	47.8	26.9	14.7	10.6
S29	0.945	51.5	20.7	13.5	14.3	0.140	53.9	26.3	9.75	10.0
Range	0.703–13.4	29.2–62.9	13.0–31.6	6.95–31.7	4.25–42.7	0.0936–4.19	37.1–73.4	18.2–40.0	4.07–20.4	4.35–25.4
Median	1.70	43.5	24.2	15.3	16.7	0.212	48.8	25.0	12.9	10.3
GM	1.96	42.4	23.0	14.7	16.2	0.259	50.1	25.9	11.3	9.92

## Data Availability

The data presented in this study are available upon request from the corresponding authors. The data are not publicity available due to the very large sizes of the chromatographic files.

## Supplementary Materials

The following supporting information can be downloaded at: https://www.mdpi.com/article/10.3390/toxics11070558/s1, Table S1: The congener groups, the percentage of Cl in molecular formula (Cl%), quantitation ion (QI, m/z), and confirmation ion (CI, m/z) for determination of 24 SCCP and 24 MCCP congeners with QI (m/z) of ^13^C_10_-trans-chlordane and hexachlorobenzene; Table S2: Comparison of SCCPs and MCCPs concentrations (ng/g dw) in marine sediments of this study with other studies; Table S3: The relative abundance for chlorine homolog group profiles (%) in marine sediments of the East China Sea and Yellow Sea; Figure S1: Spearman correlations among 24 SCCPs and 24 MCCPs in marine sediments in the East China Sea and Yellow Sea; Figure S2: The proportion of C_10_-, C_11_-, C_12_-, C_13_-congeners in SCCPs and C_14_-, C_15_-, C_16_-, C_17_-congeners in MCCPs for marine sediment in the East China Sea and Yellow Sea; Figure S3: The proportion of C_l5_-, C_l6_-, C_l7_-, C_l8_-, C_l9_-, Cl_10_-congeners in SCCPs and MCCPs for marine sediment in the East China Sea and Yellow Sea; Figure S4: The concentration of SCCPs and MCCPs in the East China Sea (in black) and Yellow Sea (in red).

## References

[1] Bayen S., Obbard J.P., Thomas G.O. (2006). Chlorinated paraffins: A review of analysis and environmental occurrence. Environ. Int..

[2] Feo M.L., Eljarrat E., Barcelo D. (2009). Occurrence, fate and analysis of polychlorinated n-alkanes in the environment. Trac-Trends Anal. Chem..

[3] Zhan F.Q., Zhang H.J., Wang J., Xu J.Z., Yuan H.P., Gao Y., Su F., Chen J.P. (2017). Release and Gas-Particle Partitioning Behaviors of Short-Chain Chlorinated Paraffins (SCCPs) During the Thermal Treatment of Polyvinyl Chloride Flooring. Environ. Sci. Technol..

[4] Gluge J., Schinkel L., Hungerbuhler K., Cariou R., Bogdal C. (2018). Environmental Risks of Medium-Chain Chlorinated Paraffins (MCCPs): A Review. Environ. Sci. Technol..

[5] Zhao N., Cui Y., Wang P.W., Li S.S., Jiang W., Luo N.N., Wang Z.H., Chen X.F., Ding L. (2019). Short-chain chlorinated paraffins in soil, sediment, and seawater in the intertidal zone of Shandong Peninsula, China: Distribution and composition. Chemosphere.

[6] Hu H.M., Qu J.L., Zhao M.R., Wu P.F., Zhu W.N., Zhou Y.D., Jin H.B. (2021). Bioaccumulation and trophic magnification of short chain chlorinated paraffins in marine organisms from East China Sea. Mar. Pollut. Bull..

[7] Zeng L.X., Chen R., Zhao Z.S., Wang T., Gao Y., Li A., Wang Y.W., Jiang G.B., Sun L.G. (2013). Spatial Distributions and Deposition Chronology of Short Chain Chlorinated Paraffins in Marine Sediments across the Chinese Bohai and Yellow Seas. Environ. Sci. Technol..

[8] Zeng L.X., Zhao Z.S., Li H.J., Wang T., Liu Q., Xiao K., Du Y.G., Wang Y.W., Jiang G.B. (2012). Distribution of Short Chain Chlorinated Paraffins in Marine Sediments of the East China Sea: Influencing Factors, Transport and Implications. Environ. Sci. Technol..

[9] Zeng L.X., Wang T., Wang P., Liu Q., Han S.L., Yuan B., Zhu N.L., Wang Y.W., Jiang G.B. (2011). Distribution and Trophic Transfer of Short-Chain Chlorinated Paraffins in an Aquatic Ecosystem Receiving Effluents from a Sewage Treatment Plant. Environ. Sci. Technol..

[10] Wang H.P., Chang H., Zhang C.X., Wu F.C. (2019). Occurrence and mass balance of medium- and long-chain chlorinated paraffins in a municipal sewage treatment plant: Comparison to short-chain compounds. Environ. Int..

[11] Gao Y., Zhang H.J., Su F., Tian Y.Z., Chen J.P. (2012). Environmental Occurrence and Distribution of Short Chain Chlorinated Paraffins in Sediments and Soils from the Liaohe River Basin, P.R. China. Environ. Sci. Technol..

[12] Pan X.H., Tang J.H., Tian C.G., Li J., Zhang G. (2018). Short- and medium-chain chlorinated paraffins in sediments from the Laizhou Bay area, North China: Implications for transportation from rivers to marine environment. Environ. Pollut..

[13] Ji B.J., Wu Y., Liang Y., Gao S.T., Zeng X.Y., Yao P., Yu Z.Q. (2022). Occurrence, congener patterns, and potential ecological risk of chlorinated paraffins in sediments of Yangtze River Estuary and adjacent East China Sea. Environ. Monit. Assess..

[14] Hussy I., Webster L., Russell M., Moffat C. (2012). Determination of chlorinated paraffins in sediments from the Firth of Clyde by gas chromatography with electron capture negative ionisation mass spectrometry and carbon skeleton analysis by gas chromatography with flame ionisation detection. Chemosphere.

[15] Chen H., Han X., Liang B.W., Deng M., Du B.B., Zeng L.X. (2022). Spatial distribution, homologue patterns and ecological risks of chlorinated paraffins in mangrove sediments along the South China Coast. Environ. Pollut..

[16] Zhao Z.S., Li H.J., Wang Y.W., Li G.L., Cao Y.L., Zeng L.X., Lan J., Wang T., Jiang G.B. (2013). Source and Migration of Short-Chain Chlorinated Paraffins in the Coastal East China Sea Using Multiproxies of Marine Organic Geochemistry. Environ. Sci. Technol..

[17] Hu H.M., Jin H.B., Li T.J., Guo Y.M., Wu P.F., Xu K.D., Zhu W.B., Zhou Y.Q., Zhao M.R. (2022). Spatial distribution, partitioning, and ecological risk of short chain chlorinated paraffins in seawater and sediment from East China Sea. Sci. Total Environ..

[18] Chen M.Y., Luo X.J., Zhang X.L., He M.J., Chen S.J., Mi B.X. (2011). Chlorinated Paraffins in Sediments from the Pearl River Delta, South China: Spatial and Temporal Distributions and Implication for Processes. Environ. Sci. Technol..

[19] Canadian Environmental Protection Act (1999). Federal Environmental Quality Guidelines Chlorinated Alkanes, May 2018.

[20] Ma X.D., Zhang H.J., Wang Z., Yao Z.W., Chen J.W., Chen J.P. (2014). Bioaccumulation and Trophic Transfer of Short Chain Chlorinated Paraffins in a Marine Food Web from Liaodong Bay, North China. Environ. Sci. Technol..

[21] Qiao L., Xia D., Gao L.R., Huang H.T., Zheng M.H. (2016). Occurrences, sources and risk assessment of short- and medium-chain chlorinated paraffins in sediments from the middle reaches of the Yellow River, China. Environ. Pollut..

[22] Wang X., Xu H., Zhou Y., Wu C., Kanchanopas-Barnette P. (2015). Distribution and source apportionment of polycyclic aromatic hydrocarbons in surface sediments from Zhoushan Archipelago and Xiangshan Harbor, East China Sea. Mar. Pollut. Bull..

[23] Wang X., Xu H., Zhou Y., Wu C., Kanchanopas-Barnette P. (2016). Spatial distribution and sources of polychlorinated biphenyls in surface sediments from the Zhoushan Archipelago and Xiangshan Harbor, East China Sea. Mar. Pollut. Bull..

