# Supplementary material for: Short- and Medium-Chain Chlorinated Paraffins in the Sediment of the East China Sea and Yellow Sea: Distribution, Composition, and Ecological Risks
Source: Toxics. 2023 Jun 26;11(7):558. doi: 10.3390/toxics11070558 (PMC10383561; doi:10.3390/toxics11070558)
Supplement: Supplementary file 1 [file toxics-11-00558-s001.zip › toxics-2450314-supplementary.pdf]

Short- and Medium-Chain Chlorinated Paraffins in the Sediment of the East China Sea and Yellow Sea: Distribution, Composition, and Ecological Risks

Xiaoying Li [1], Haiqiang Guo [1], Jianyao Hong [1,2], Yuan Gao [2,*], Xindong Ma [3] and Jiping Chen [2]

[1] College of Environmental Science and Engineering, Dalian Maritime University, Dalian 116026, China

[2] CAS Key Laboratory of Separation Sciences for Analytical Chemistry, Dalian Institute of Chemical Physics, Chinese Academy of Sciences, 457 Zhongshan Road, Shahekou District, Dalian 116023, China

[3] State Environmental Protection Key Laboratory of Coastal Ecosystem, National Marine Environmental Monitoring Center, Dalian 116023, China

* Correspondence: acyoyo@dicp.ac.cn; Tel.: +86-411-8437-9972; Fax: +86-411-8437-9562

Tables and Figures

Tables:

Table S1. The congener groups, the percentage of Cl in molecular formula (Cl%), quantitation ion (QI, m/z), and confirmation ion (CI, m/z) for determination of 24 SCCP and 24 MCCP congeners with QI (m/z) of $^{13}C_{10}$-trans-chlordane and hexachlorobenzene.

Table S2. Comparison of SCCPs and MCCPs concentrations (ng/g dw) in marine sediments of this study with other studies.

Table S3. The relative abundance for chlorine homolog group profiles (%) in marine sediments of the East China Sea and Yellow Sea.

Figures:

Figure S1. Spearman correlations among 24 SCCPs and 24 MCCPs in marine sediments in the East China Sea and Yellow Sea.

Figure S2. The proportion of C_{10}-, C_{11}-, C_{12}-, C_{13}-congeners in SCCPs and C_{14}-, C_{15}-, C_{16}-, C_{17}-congeners in MCCPs for marine sediment in the East China Sea and Yellow Sea.

Figure S3. The proportion of Cl_5-, Cl_6-, Cl_7-, Cl_8-, Cl_9-, Cl_{10}-congeners in SCCPs and MCCPs for marine sediment in the East China Sea and Yellow Sea.

Figure S4. The concentration of SCCPs and MCCPs in the East China Sea (in black) and Yellow Sea (in red).

Table S1. The congener groups, the percentage of Cl in molecular formula (Cl%), quantitation ion (QI, m/z), and confirmation ion (CI, m/z) for determination of 24 SCCP and 24 MCCP congeners with QI (m/z) of $^{13}C_{10}$-trans-chlordane and hexachlorobenzene.

**SCCPs**	Cl%	QI (m/z)	CI (m/z)	**MCCPs**	Cl%	QI (m/z)	CI (m/z)
$C_{10}H_{17}Cl_5$	56.4	279	277	$C_{14}H_{25}Cl_5$	47.8	333	335
$C_{10}H_{16}Cl_6$	61.0	313	315	$C_{14}H_{24}Cl_6$	52.5	369	371
$C_{10}H_{15}Cl_7$	64.7	347	349	$C_{14}H_{23}Cl_7$	56.5	403	405
$C_{10}H_{14}Cl_8$	67.9	381	383	$C_{14}H_{22}Cl_8$	59.8	437	439
$C_{10}H_{13}Cl_9$	70.6	417	415	$C_{14}H_{21}Cl_9$	62.8	473	471
$C_{10}H_{12}Cl_{10}$	72.8	451	449	$C_{14}H_{20}Cl_{10}$	65.3	505	507
$C_{11}H_{19}Cl_5$	54.3	293	291	$C_{15}H_{27}Cl_5$	46.1	349	347
$C_{11}H_{18}Cl_6$	58.9	327	329	$C_{15}H_{26}Cl_6$	50.8	383	385
$C_{11}H_{17}Cl_7$	62.8	361	363	$C_{15}H_{25}Cl_7$	54.7	417	419
$C_{11}H_{16}Cl_8$	66.0	395	397	$C_{15}H_{24}Cl_8$	58.1	451	453
$C_{11}H_{15}Cl_9$	68.7	431	429	$C_{15}H_{23}Cl_9$	61.1	485	487
$C_{11}H_{14}Cl_{10}$	71.1	465	463	$C_{15}H_{22}Cl_{10}$	63.7	521	519
$C_{12}H_{21}Cl_5$	52.4	307	305	$C_{16}H_{29}Cl_5$	44.5	361	363
$C_{12}H_{20}Cl_6$	57.0	341	343	$C_{16}H_{28}Cl_6$	49.1	397	399
$C_{12}H_{19}Cl_7$	60.9	375	377	$C_{16}H_{27}Cl_7$	53.1	431	433
$C_{12}H_{18}Cl_8$	64.2	409	411	$C_{16}H_{26}Cl_8$	56.5	465	467
$C_{12}H_{17}Cl_9$	67.0	445	443	$C_{16}H_{25}Cl_9$	59.5	501	499
$C_{12}H_{16}Cl_{10}$	69.4	479	477	$C_{16}H_{24}Cl_{10}$	62.1	535	533
$C_{13}H_{23}Cl_5$	50.6	321	319	$C_{17}H_{31}Cl_5$	43.0	375	377
$C_{13}H_{22}Cl_6$	55.3	355	357	$C_{17}H_{30}Cl_6$	47.6	411	413
$C_{13}H_{21}Cl_7$	59.2	389	391	$C_{17}H_{29}Cl_7$	51.5	443	445
$C_{13}H_{20}Cl_8$	62.5	423	425	$C_{17}H_{28}Cl_8$	55.0	477	479

$C_{13}H_{19}Cl_9$	65.3	459	457	$C_{17}H_{27}Cl_9$	58.0	515	513
$C_{13}H_{18}Cl_{10}$	67.8	493	491	$C_{17}H_{26}Cl_{10}$	60.6	549	547
$^{13}C_{10}$-trans-chlordane		419.8		Hexachlorobenzene		284	

Table S2. Comparison of SCCPs and MCCPs concentrations (ng/g dw) in marine sediments of this study with other studies.

Sampling Year	Sampling cities	Number of samples	The thickness of sediment	SCCPs					MCCPs				
				DF	GM	Median	Mean	Range	DF	GM	Median	Mean	Range
2019	East China Sea and Yellow Sea (this study)	29	Surface sediment (0-5 cm)	100%	1.96	1.70	2.55	0.703–13.4	100%	0.259	0.212	0.468	0.0936–4.19
2009 [1]	Laizhou Bay	29	Surface sediment	100%	10.4	10.2	11.1	5.10–22.0	100%	6.11	5.55	9.65	2.20–63.0
2017 [2]	Yangtze River Estuary and adjacent East China Sea	87	Surface sediment (0-5 cm)	100%	NP	13.8	18.6	2.85–94.7	100%	NP	13.3	18.5	3.33–77.8
2007 [3]	Firth of Clyde in Scotland	11	Surface sediment (0-5 cm)	100%	15.3	31.0	34.3	0.40–69.0	NP	NP	NP	NP	NP
2017 [4]	Shandong Peninsula	87	Intertidal sediment	96.6%	89.1	82.2	117	<MDL–453	NA	NA	NA	NA	NA
2017–2019 [5]	the South China Coast (Guangdong, Fujian, Guangxi, and Hainan)	72	Mangrove sediments	100%	345	331	371	133–716	100%	647	666	937	103–4160
2010–2011 [6]	East China Sea	37	Surface sediment (0-3 cm)	100%	22.7	24.7	24.0	9.00–37.2	NA	NA	NA	NA	NA
2019 [7]	East China Sea	40	Surface sediment (0-3 cm)	100%	NP	189	194	89.6–351	NA	NA	NA	NA	NA

2009–2010 [8]	Pearl River Estuary	13	Estuarine sediments	NP	NP	NP	1700	NP	NP	NP	NP	NP	NP

DF: The detection frequency. GM: Geometric mean. NP. Not provided in previous study. NA: Not analyzed.

Table S3. The relative abundance for chlorine homolog group profiles (%) in marine sediments of the East China Sea and Yellow Sea.

Sampling sites	SCCPs (%)						MCCPs (%)					
	Cl_5	Cl_6	Cl_7	Cl_8	Cl_9	Cl_{10}	Cl_5	C_6	Cl_7	Cl_8	Cl_9	Cl_{10}
S1	1.13	7.42	19.8	27.2	27.3	17.0	8.54	16.4	25.9	30.3	14.7	4.08
S2	8.32	27.5	21.5	20.3	14.4	7.90	14.5	16.5	31.8	27.0	8.23	1.94
S3	8.39	30.0	20.0	20.4	15.3	5.92	20.8	22.9	26.0	22.4	6.59	1.41
S4	10.4	30.7	18.8	20.0	14.6	5.57	17.5	22.5	27.1	24.5	7.12	1.18
S5	3.73	12.9	19.7	25.5	27.3	10.7	5.18	14.8	32.4	36.8	9.73	1.13
S6	6.57	25.3	28.5	24.2	10.6	4.79	22.3	24.1	22.9	21.2	7.82	1.77
S7	12.7	32.8	21.7	16.7	10.3	5.88	19.1	20.5	24.6	24.2	9.60	1.99
S8	15.1	34.7	19.9	13.7	10.6	6.02	19.4	17.4	25.6	25.7	10.2	1.64
S9	8.13	36.2	29.3	14.1	8.10	4.15	32.6	21.9	19.6	18.2	6.05	1.71
S10	7.27	17.3	14.2	37.6	18.2	5.51	9.11	18.9	36.6	25.1	8.71	1.59
S11	14.8	34.3	18.5	15.3	11.3	5.75	16.7	16.9	25.2	26.7	11.9	2.54
S12	25.0	51.4	18.2	3.15	1.32	0.870	61.5	16.0	9.95	8.26	3.04	1.28
S13	8.56	26.7	23.1	25.4	12.7	3.63	33.9	16.5	19.2	19.5	9.12	1.70
S14	16.4	47.3	22.0	6.85	4.94	2.55	35.1	20.8	21.5	14.0	6.73	1.75
S15	10.8	35.3	28.2	15.2	7.33	3.16	29.1	30.1	20.1	13.8	5.47	1.45
S16	15.0	39.9	22.1	12.2	6.86	3.92	29.8	24.9	21.4	16.4	5.08	2.47
S17	14.9	49.1	25.3	6.70	2.80	1.20	49.5	20.2	13.6	11.5	3.65	1.54
S18	7.08	30.3	23.5	16.7	15.9	6.52	14.4	16.9	27.5	30.5	9.24	1.53
S19	15.2	39.4	27.9	10.2	4.25	3.09	41.1	27.5	13.8	11.3	4.28	2.07
S20	16.9	41.5	26.9	7.68	3.98	3.14	41.3	31.7	11.7	8.22	5.13	1.91
S21	14.0	36.1	28.6	11.1	6.79	3.43	28.2	31.5	18.4	15.2	5.28	1.33
S22	7.56	25.8	28.7	17.9	14.0	6.03	11.8	27.2	29.4	23.1	7.46	1.15

S23	15.0	34.9	28.2	10.0	7.64	4.22	26.0	28.1	19.5	17.0	7.22	2.16
S24	14.3	41.4	25.5	10.8	5.20	2.70	34.4	21.5	18.9	17.1	6.62	1.51
S25	4.97	18.6	26.3	23.7	19.1	7.31	6.89	24.0	31.7	26.8	9.18	1.49
S26	8.51	30.3	26.9	17.6	11.0	5.77	15.1	26.1	26.7	22.5	8.09	1.55
S27	15.2	42.5	24.1	9.07	6.01	3.14	25.3	25.7	21.6	18.8	7.20	1.41
S28	11.1	38.0	29.7	11.0	6.45	3.79	30.7	30.4	17.2	14.0	5.88	1.81
S29	12.4	37.0	22.4	12.8	9.25	6.09	23.2	19.5	21.3	22.3	10.3	3.33

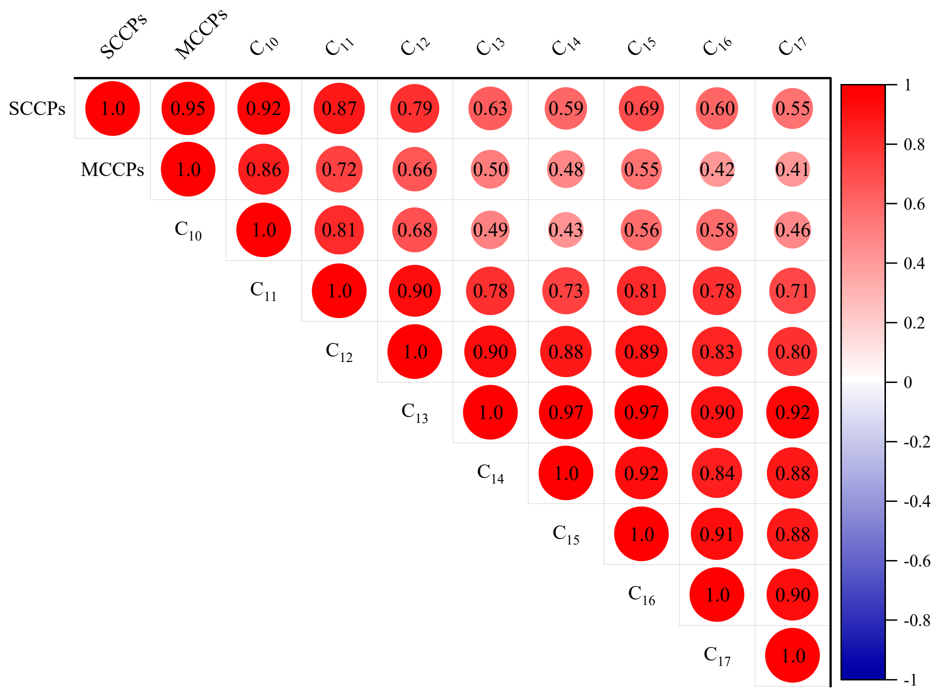

Figure S1. Spearman correlations among 24 SCCPs and 24 MCCPs in marine sediments in the East China Sea and Yellow Sea.

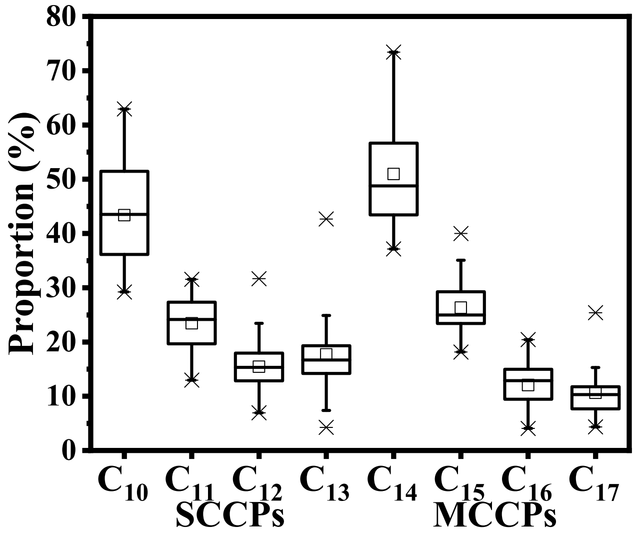

Figure S2. The proportion of C_{10}-, C_{11}-, C_{12}-, C_{13}-congeners in SCCPs and C_{14}-, C_{15}-, C_{16}-, C_{17}-congeners in MCCPs for marine sediment in the East China Sea and Yellow Sea.

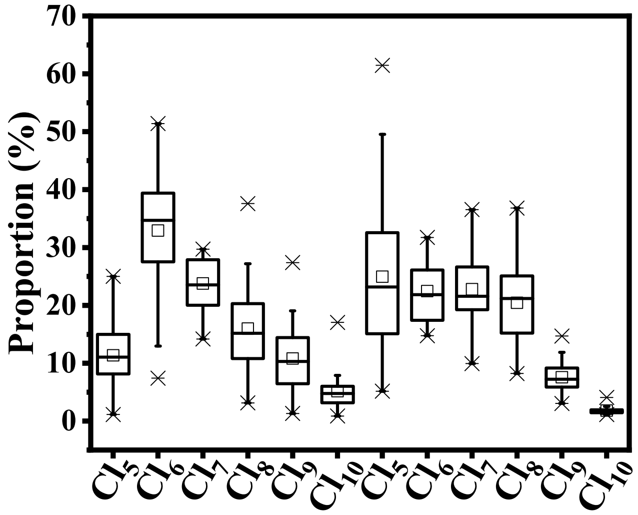

Figure S3. The proportion of Cl_5-, Cl_6-, Cl_7-, Cl_8-, Cl_9-, Cl_{10}-congeners in SCCPs and MCCPs for marine sediment in the East China Sea and Yellow Sea.

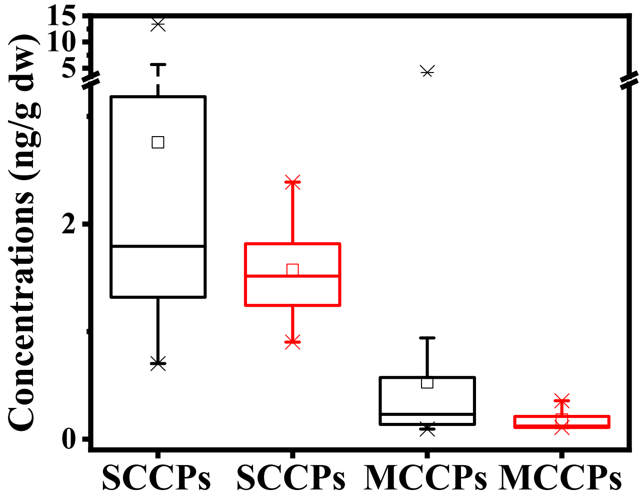

Figure S4. The concentration of SCCPs and MCCPs in the East China Sea (in black) and Yellow Sea (in red).

References

1. Pan, X.H.; Tang, J.H.; Tian, C.G.; Li, J.; Zhang, G. Short- and medium-chain chlorinated paraffins in sediments from the Laizhou Bay area, North China: Implications for transportation from rivers to marine environment. *Environmental Pollution* **2018**, *243*, 1460-1468, doi:10.1016/j.envpol.2018.09.123.
2. Ji, B.J.; Wu, Y.; Liang, Y.; Gao, S.T.; Zeng, X.Y.; Yao, P.; Yu, Z.Q. Occurrence, congener patterns, and potential ecological risk of chlorinated paraffins in sediments of Yangtze River Estuary and adjacent East China Sea. *Environmental Monitoring and Assessment* **2022**, *194*, 329, doi:10.1007/s10661-022-09969-8.
3. Hussy, I.; Webster, L.; Russell, M.; Moffat, C. Determination of chlorinated paraffins in sediments from the Firth of Clyde by gas chromatography with electron capture negative ionisation mass spectrometry and carbon skeleton analysis by gas chromatography with flame ionisation detection. *Chemosphere* **2012**, *88*, 292-299, doi:10.1016/j.chemosphere.2012.02.040.
4. Zhao, N.; Cui, Y.; Wang, P.W.; Li, S.S.; Jiang, W.; Luo, N.N.; Wang, Z.H.; Chen, X.F.; Ding, L. Short-chain chlorinated paraffins in soil, sediment, and seawater in the intertidal zone of Shandong Peninsula, China: Distribution and composition. *Chemosphere* **2019**, *220*, 452-458, doi:10.1016/j.chemosphere.2018.12.063.
5. Chen, H.; Han, X.; Liang, B.W.; Deng, M.; Du, B.B.; Zeng, L.X. Spatial distribution, homologue patterns and ecological risks of chlorinated paraffins in mangrove sediments along the South China Coast. *Environmental Pollution* **2022**, *294*, 118623, doi:10.1016/j.envpol.2021.118623.
6. Zhao, Z.S.; Li, H.J.; Wang, Y.W.; Li, G.L.; Cao, Y.L.; Zeng, L.X.; Lan, J.; Wang, T.; Jiang, G.B. Source and Migration of Short-Chain Chlorinated Paraffins in the Coastal East China Sea Using Multiproxies of Marine Organic Geochemistry. *Environmental Science & Technology* **2013**, *47*, 5013-5022, doi:10.1021/es304899j.
7. Hu, H.M.; Jin, H.B.; Li, T.J.; Guo, Y.M.; Wu, P.F.; Xu, K.D.; Zhu, W.B.; Zhou, Y.Q.; Zhao, M.R. Spatial distribution, partitioning, and ecological risk of short chain chlorinated paraffins in seawater and sediment from East China Sea. *Science of the Total Environment* **2022**, *811*, 151932, doi:10.1016/j.scitotenv.2021.151932.
8. Chen, M.Y.; Luo, X.J.; Zhang, X.L.; He, M.J.; Chen, S.J.; Mi, B.X. Chlorinated Paraffins in Sediments from the Pearl River Delta, South China: Spatial and Temporal Distributions and Implication for Processes. *Environmental Science & Technology* **2011**, *45*, 9936-9943, doi:10.1021/es202891a.
